# Of attachment and connection: Auxin signaling in the cambium promotes successful plant grafting

**DOI:** 10.1093/plphys/kiae334

**Published:** 2024-06-12

**Authors:** Janlo M Robil

**Affiliations:** Assistant Features Editor, Plant Physiology, American Society of Plant Biologists; Department of Biology, School of Science and Engineering, Ateneo de Manila University, Quezon City 1108, Philippines

Plants possess an extraordinarily regenerative ability that allows excised shoot and root systems of two plants to be joined and grow as one. This technique, known as grafting, has been practiced for millennia to improve performance and yields in many crops by combining advantageous traits of the shoot (scion) with those of the root system (rootstock) (Williams et al. 2021). Successful grafting depends on the attachment of tissues, the formation of an unspecialized mass of cells called callus, and the connection of vascular tissues between the grafted organs (Melnyk et al. 2015). The signal triggering these processes was thought to originate from the pericycle, the meristematic outer cell layer of the vascular cylinder (Iwase et al. 2011). The cambium, the stem cell niche that produces vascular cell precursors, is also hypothesized to regulate grafting, as mutations in cambium-associated genes reduce grafting efficiency (Matsuoka et al. 2021; Zhang et al. 2022). However, the role of cambium in graft formation has not yet been experimentally tested.

**Figure 1.**
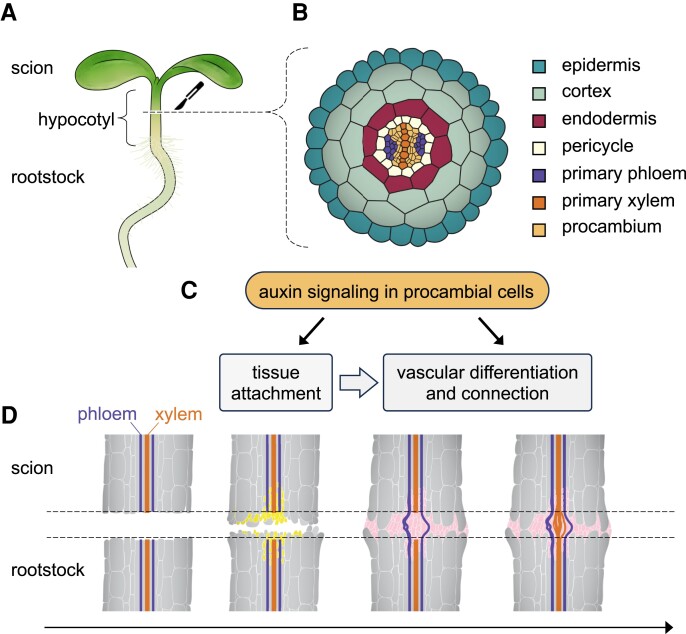
Auxin signaling in the cambium plays an important role during plant grafting. **A)** Diagram of a grafted Arabidopsis seedling, showing the scion (top) and rootstock (bottom) excised and joined at the hypocotyl. **B)** Transverse sectional view of an Arabidopsis hypocotyl, illustrating the tissue arrangement. **C)** A schematic representation of the role of auxin signaling in procambial cells, highlighting its functions in tissue attachment and vascular differentiation and connection during graft formation (Serivichyaswat et al. 2024). **D)** An illustration of longitudinal sections of scion and rootstock showing the stages of graft formation, with the broken lines indicating the graft interface. Cell proliferation (yellow) and cell expansion facilitate tissue attachment and callus formation (pink), leading to the reconnection of phloem and xylem.

In most eudicots, the stem and root tissues are arranged in a concentric pattern, wherein the epidermis and ground tissues surround the vascular cylinder. Within this cylinder, the cambium is positioned between the inner xylem and outer phloem (Fig. 1, A and B). During early seedling development, the cambium forms through the longitudinal division of procambial cells. The plant hormone auxin plays a crucial role in controlling cambial cell division and vascular cell differentiation. Notably, blocking auxin signaling prevents grafting, though specific cell-type responses remain unclear (Melnyk et al. 2015). In this issue of *Plant Physiology*, Serivichyaswat et al. (2024) explored the role of auxin signaling in individual cell types during graft formation in Arabidopsis. They discovered that auxin signaling in the cambium facilitates tissue attachment and vascular connection by promoting cell division and expansion in the graft union.

To dissect the spatial-temporal auxin response during graft formation, Serivichyaswat et al. (2024) used an inducible cell-specific expression system to “switch off” auxin signaling in specific cell types. Normally, in the presence of auxin, AUX/IAAs are degraded, freeing ARFs to induce gene expression. However, the authors used a mutant version of the AUX/IAA protein BODENLOS (BDL) (Hamann et al. 2002), which cannot be degraded, thereby blocking auxin signaling.

The expression system comprises an effector line and a driver line (Schürholz et al. 2018). The effector line contains the mutant *bdl* under the control of a synthetic promoter that is activated only by a specific transcription factor. This line was crossed with 11 driver lines, each having cell-type-specific promoters driving the expression of the transcription factor and a GFP in an inducible manner. Upon chemical induction, the resulting driver/effector F_1_ line misexpressed *bdl* and produced a fluorescent signal in specific tissues, such as the epidermis, endodermis, pericycle cells, phloem, vascular cell precursors, and procambium (Fig. 1B). This *bdl* misexpression system enabled the authors to both visualize transgene expression and selectively block auxin signaling, thus decoupling cell-type responses during graft formation (Serivichyaswat et al. 2024).

How does blocking auxin signaling in different cell types affect grafting? Using the *bdl* misexpression lines, Serivichyaswat et al. (2024) conducted grafting experiments and assessed tissue attachment and phloem reconnection in the graft junctions. Plants that expressed *bdl* in the procambium failed to attach, form callus, and reconnect vascular tissues. Additionally, *bdl* expression in the procambium and vascular precursors specifically inhibited phloem connection. These effects were not unique to *bdl*, as similar results were observed when a different dominant AUX/IAA mutant was misexpressed in these cell types (Serivichyaswat et al. 2024). These findings suggest that auxin signaling in the cambium is crucial for successful graft formation.

It is known that the scion and rootstock play different roles in graft formation (Melnyk et al. 2015). Thus, the authors investigated whether auxin signaling in the scion, the rootstock, or both is crucial to the union. They performed grafts with combinations of scions and rootstocks either expressing only the driver (normal auxin signaling) or the driver plus the effector (auxin signaling “off”). Plants expressing *bdl* driven by procambial cell-specific promoters in various scion and rootstock combinations exhibited varied graft responses. In addition, confocal microscopy revealed overlapping but also distinct expression patterns of these promoters in the graft junctions. Therefore, the authors posit that subsets of procambial cells may play distinct roles in tissue attachment and vascular connection, with the shoot and root systems potentially exerting specific influences.

Proliferation and expansion of cells in the cut sites are critical early responses during grafting as they facilitate attachment and callus establishment (Jeffree and Yeoman 2006). Could the misexpression of *bdl* in the cambium affect these cellular processes? Using EdU (5-ethynyl-2′-deoxyuridine) staining to detect cell division, the authors demonstrated that graft junctions of plants expressing *bdl* in the procambium showed no indication of cell proliferation compared to normal grafts. Furthermore, in cut but ungrafted *bdl* expressing plants, the cut sites exhibited little cell proliferation and expansion. These findings indicate that auxin signaling in the cambium could be driving callus formation and vascular connection by promoting cell division and expansion in the cut sites. Since cambial mutants have displayed impaired graft formation (Serivichyaswat et al. 2024), clarifying how the cambium regulates these processes remains an important question for future study.

In summary, Serivichyaswat et al. (2024) employed an inducible cell-specific expression system to investigate the roles of individual cell types in graft formation. By misexpressing *bdl*, they demonstrated the importance of auxin signaling in the cambium and revealed previously unknown roles of procambial cells in facilitating tissue attachment and vascular connection (Fig. 1, C and D). This study could help explain why monocots, which lack cambium in their mature stems, fail to graft but can do so successfully at their embryonic stages (Reeves et al. 2022). A more comprehensive understanding of cambium-derived signaling mechanisms could significantly enhance efforts to graft important perennial crops and leverage plants' regenerative ability to cultivate more resilient varieties.
